# Ambulatory Holter Findings in Patients with Palpitations and Structurally Normal Heart: A Prospective Study of the Prevalence and Patterns of Ventricular and Supraventricular Arrhythmias

**DOI:** 10.3390/jcm15093285

**Published:** 2026-04-25

**Authors:** Khaled Elenizi, Rasha Alharthi, Nasser E. Alotaibi, Talal Alotaibi, Mohammed Alfraikh, Faris Almusayfir, Kamran Ahmad

**Affiliations:** 1 Department of Internal Medicine, College of Medicine, Prince Sattam bin Abdulaziz University, Al-Kharj 11942, Saudi Arabia; 2 Department of Cardiology, Al-Iman General Hospital, Riyadh 12614, Saudi Arabia; 3 College of Medicine, Prince Sattam bin Abdulaziz University, Al-Kharj 11942, Saudi Arabia; 4 Department of Cardiology, Al Salam Health Medical Hospital, Riyadh 14224, Saudi Arabia

**Keywords:** Holter monitoring, palpitations, premature ventricular contractions, atrial premature contractions, heart rate variability, Saudi Arabia

## Abstract

**Background/Objectives:** Palpitations are among the most common reasons for cardiology referrals. Despite widespread use of ambulatory cardiac monitoring, contemporary data from the Middle East are scarce. Extended Holter monitoring provides detailed evaluation of arrhythmia burden, autonomic regulation, and symptom–rhythm correlation in routine clinical practice. **Methods:** We conducted a prospective observational study of consecutive patients presenting with palpitations who underwent 24–96 h ambulatory Holter monitoring at a cardiology outpatient clinic in Saudi Arabia in 2025. Demographic and clinical characteristics, comorbidities, medication use, echocardiographic parameters, heart rate variability (HRV), ventricular and supraventricular ectopy, tachyarrhythmias, and symptom diary activations were systematically evaluated. **Results:** Among 251 patients (mean age 41.9 ± 16.4 years; 35.5% male), Holter monitoring showed excellent recording quality (mean analyzable time 98.7 ± 9.5%). Premature ventricular contractions (PVCs) were detected in 53.4% of patients, but burden was low (median 0.0%, IQR 0–0.1%), with only 4.4% exceeding 10%. Atrial premature contractions (APCs) were common (92.0%), though usually low-burden (median burden 0.0%, IQR 0–0.1%); atrial fibrillation and supraventricular tachycardia were rare (0.8% each). Symptom diary activation occurred in 116 patients (46.2%), with 996 events; most (87.9%) correlated with sinus tachycardia, while only 8.6% correlated with PVCs and 2.6% with APCs. In the remaining 53.8% of patients, no symptom–rhythm correlation was documented during monitoring. Heart rate variability showed expected age-related changes. **Conclusions:** In this predominantly young cohort, Holter monitoring revealed frequent low-burden atrial and ventricular ectopy, whereas clinically significant tachyarrhythmias were uncommon. Holter monitoring up to 96 h provided a diagnostic yield in approximately 50% of patients and should be considered a first-line screening tool. Patients without diagnostic findings may require prolonged monitoring using external or implantable devices.

## 1. Introduction

Palpitations are among the most common reasons for cardiology referral [1], yet contemporary real-world data from the Middle East remain limited. Ambulatory Holter monitoring is a cornerstone of evaluation for intermittent symptoms not captured on resting electrocardiogram (ECG), enabling continuous rhythm assessment with enhanced detection of premature ventricular contractions (PVCs), atrial premature contractions (APCs), supraventricular and ventricular tachyarrhythmias, and heart rate variability (HRV) metrics [2,3]. PVCs are frequently detected on ambulatory monitoring [4]. PVC prevalence is defined as the presence of ≥1 PVC during monitoring ranges from approximately 0.8–5.5% on resting ECG, increasing substantially with age, cardiovascular risk factors, and longer-duration ambulatory monitoring. In Holter-based studies, the prevalence of PVCs ranges from 40% to 75% in healthy adults or individuals without known structural heart disease, often exceeding 60% with extended monitoring or in older cohorts [5,6]. APCs are often considered benign, but higher APC burden has been associated with subsequent atrial fibrillation (AF) and stroke risk in longitudinal studies [7,8]. Beyond arrhythmia detection, HRV reflects autonomic modulation and reduced HRV, particularly in long-term measures such as standard deviation of normal-to-normal intervals (SDNN) and standard deviation of the average normal-to-normal intervals (SDANN), and has been linked with aging and adverse cardiovascular outcomes [9]. Current guidelines from major cardiovascular societies highlight the role of ambulatory rhythm monitoring in symptomatic patients, emphasizing that extended recording durations improve arrhythmia detection, symptom–rhythm concordance, and clinical decision-making [2,10,11]. In this prospective observational study, we characterized the prevalence, burden, and clinical correlates of arrhythmias in a contemporary Middle Eastern cohort referred for palpitations. Using extended Holter monitoring in 251 patients, we analyzed ventricular and supraventricular ectopy, arrhythmia patterns (including couplets, bigeminy, and nonsustained ventricular tachycardia [NSVT]), and time-domain HRV indices. Associations with demographic characteristics, cardiovascular risk factors, medication use, and echocardiographic parameters were systematically examined. Additionally, we assessed symptom diary activations to evaluate symptom–rhythm relationships in routine clinical practice. Through combined analysis of arrhythmic, autonomic, and clinical variables, this study aims to refine individualized management strategies for patients presenting with palpitations. In particular, we emphasized symptom–rhythm correlation using patient-activated diaries and evaluated the implications of diagnostic versus non-diagnostic Holter findings for the need of prolonged monitoring.

## 2. Materials and Methods

### 2.1. Study Design and Population

This prospective observational study, conducted in accordance with The Strengthening the Reporting of Observational Studies in Epidemiology (STROBE) guidelines [12], included 12 consecutive patients aged ≥14 years presenting with palpitations who underwent ambulatory Holter monitoring at the cardiology outpatient clinic of Prince Sattam University Hospital between June and December 2025. Inclusion criteria required ≥24 h of analyzable Holter data and complete core demographic information. Exclusion criteria included patients with structural heart disease confirmed by routine baseline transthoracic echocardiography or previously documented in medical records, patients with complaints of symptoms occurring less frequently than every 4 days, incomplete recordings, and missing patient’s data. Missing variables were imputed using validated methods, with body mass index (BMI) derived from height and weight. Transthoracic echocardiography was performed as part of routine clinical evaluation prior to or contemporaneous with Holter monitoring. Holter monitoring duration was individualized according to symptom frequency (24 h for daily symptoms, 48–72 h for intermittent symptoms, and up to 96 h for less frequent symptoms). All recordings were fully analyzed and independently reviewed by a cardiac electrophysiologist (K.E.). Holter monitoring was performed during usual medical therapy; concomitant medications were prescribed for non-arrhythmic indications. All recordings were systematically screened for ventricular and supraventricular arrhythmias, conduction disturbances, pauses, and malignant ventricular rhythms; second-degree atrioventricular block, when present, was limited to Mobitz I (Wenckebach) physiology. Additional symptoms were extracted from electronic medical records but were not systematically quantified beyond the symptom diary recorded during Holter monitoring, which focused on palpitations. All participants had structurally normal hearts, preserved systolic function, and no more than non-significant valvular disease. Symptom–rhythm correlation was primarily assessed through patient-activated symptom diaries recorded during the Holter monitoring period. Prespecified study endpoints included detection and burden of PVCs, premature atrial contractions (PACs), supraventricular tachycardia (SVT), AF, and symptom–rhythm correlation. The study was approved by the Institutional Review Board of Prince Sattam bin Abdulaziz University (IRB No. SCBR-253/2024).

### 2.2. Definitions

Arrhythmias, conduction abnormalities, heart rate variability indices, and ectopy metrics were defined according to established guideline-based electrophysiology criteria, with detailed definitions and reference sources provided in Supplementary Table S1.

### 2.3. Data Collection

Holter monitoring was conducted using the Philips Zymed DigiTrak XT system (Philips Healthcare, Andover, MA, USA; Holter analysis software version 3.0.4), providing continuous electrocardiographic recording with automated arrhythmia detection followed by manual verification. Data were extracted from electronic medical records and Holter reports using standardized quality control procedures. Recordings with excessive artifacts or <24 h of analyzable data were excluded from analysis. Patients with missing core Holter data or key demographic variables were excluded from the analysis. Missing values in secondary variables were handled using multiple imputation by chained equations when appropriate. Collected variables included demographic and anthropometric data, cardiovascular risk factors and comorbidities, clinical characteristics, medication use, detailed Holter-derived rhythm and HRV metrics, symptom–rhythm correlation, and laboratory parameters.

### 2.4. Statistical Analysis

The statistical analysis described cardiac monitoring performance, heart rate patterns, and signal quality in the dataset. We summarized continuous variables, including total QRS count, heart rate measures, monitoring duration, analyzable time, and the percentage of analyzable recording time, using standard descriptive statistics such as mean, median, standard deviation, and interquartile range. We used histograms, kernel density estimates, and box-and-whisker plots to visually check for skewness, outliers, and multiple peaks in the data. To assess signal quality, we calculated the proportion of recordings with at least 99% analyzable data and highlighted these cases for subgroup comparisons. We compared recordings that met or did not meet the 99% threshold using boxplots and scatterplots to examine associations with monitoring duration, heart rate variability, and QRS burden. We used Pearson correlation coefficients for continuous variables when the data were roughly normal and linear, and Spearman’s rho for variables that were not normally distributed. Scatterplots with regression lines helped us visualize relationships, such as those between monitoring duration and analyzable time, and between analyzable percentage and QRS count. We checked the normality of residuals with Q–Q plots when needed. The analysis was primarily descriptive with exploratory associations using appropriate statistical tests, in line with standards for evaluating cardiac monitoring devices. We considered *p*-values below 0.05 as statistically significant. All analyses were done using SPSS version 26 (IBM Corp, Armonk, NY, USA).

## 3. Results

**Figure 1:**
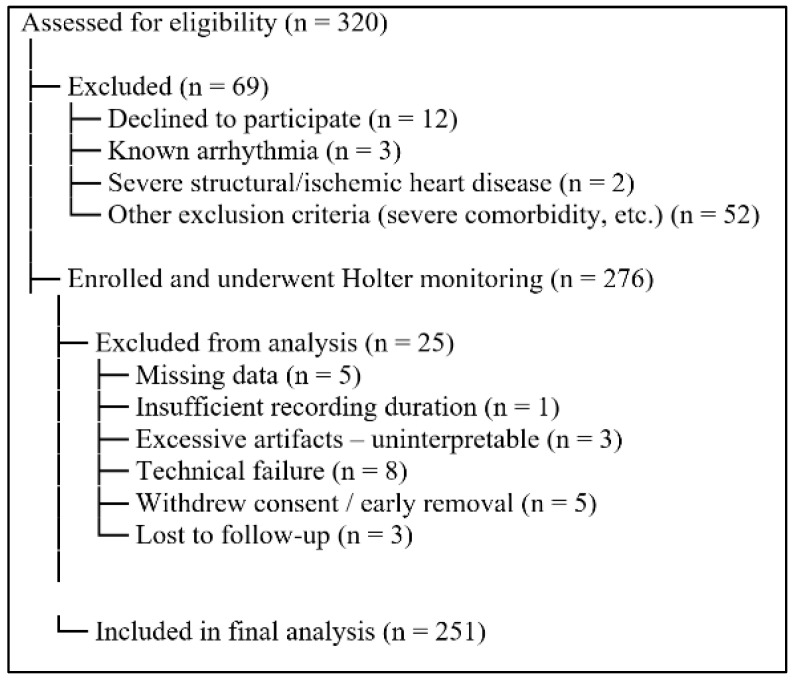
Study Flow Diagram.

Of 320 individuals screened for eligibility, 69 were excluded due to predefined exclusion criteria or refusal to participate. A total of 276 participants underwent ambulatory Holter monitoring; of these, 25 were excluded because of missing or inadequate data, technical failure, insufficient recording duration, withdrawal of consent, or loss to follow-up. The final analytical cohort comprised 251 participants (Figure 1).

### 3.1. Baseline Demographic and Clinical Characteristics

The mean age of the study population was 41.9 ± 16.4 years, with 47.8% ≤ 40 years and 52.2% > 40 years. Males comprised 35.5% of the cohort. Mean BMI was 29.5 ± 6.49 kg/m^2^, with obesity (BMI ≥ 30) in 45.0%. Mean systolic and diastolic blood pressures were 126.4 ± 14.7 mmHg and 76.2 ± 9.76 mmHg, respectively. Current smoking was uncommon (4.8%).

Common cardiometabolic comorbidities included hypertension (54.2%), dyslipidemia (32.3%), and diabetes mellitus (27.5%). Established coronary artery disease was rare (3.6%). Other notable comorbidities were COPD/asthma (12.7%), anemia (13.9%), hypothyroidism (13.9%), and anxiety/depression (6.8%) (Table 1).

### 3.2. Baseline Laboratory Findings

Laboratory parameters showed overall stability. Mean hemoglobin was 13.2 ± 1.78 g/dL. Thyroid function tests were largely normal (TSH 2.54 ± 3.99 mIU/L, FT4 15.6 ± 3.26 pmol/L, FT3 4.76 ± 1.39 pmol/L). Lipid profile: LDL-C 2.77 ± 0.94 mmol/L, HDL-C 1.39 ± 0.34 mmol/L, triglycerides 1.19 ± 0.57 mmol/L. Glycemic control was preserved (HbA1c 5.83 ± 1.20%, fasting glucose 5.89 ± 1.67 mmol/L). Renal function was normal (creatinine 68.2 ± 21.7 µmol/L) (Table 2).

### 3.3. Echocardiographic Characteristics

Left ventricular systolic function was preserved (mean LVEF 66.6 ± 5.19%). Chamber sizes and wall thicknesses were within normal limits (LVEDD 46.5 ± 4.52 mm, left atrial diameter 30.7 ± 4.88 mm). Right ventricular function was also preserved (TAPSE 21.6 ± 2.37 mm) (Table 3).

### 3.4. Baseline Medical Therapy

At baseline, 16.7% were on beta-blockers, 21.9% on statins, 9.6% on ACEi/ARBs, 3.6% on diuretics, and 2.4% on oral anticoagulants (Table 4).

### 3.5. Holter Monitoring Quality and Heart Rate Characteristics

Mean analyzable recording time was 43.5 ± 15.2 h (98.7 ± 9.53% of total time). Mean heart rate was 77.9 ± 10.4 bpm (maximum 135.2 ± 21.4, minimum 51.8 ± 8.56 bpm). Most participants had 48 h monitoring (62.5%) (Table 5).

### 3.6. Heart Rate Variability

Time-domain HRV parameters showed mean SDNN 126.6 ± 37.8 ms, RMSSD 53.0 ± 41.7 ms, ASDNN 59.9 ± 24.3 ms, and SDANN 107.0 ± 35.7 ms (Table 6).

### 3.7. Ventricular Ectopy

**Figure 2:**
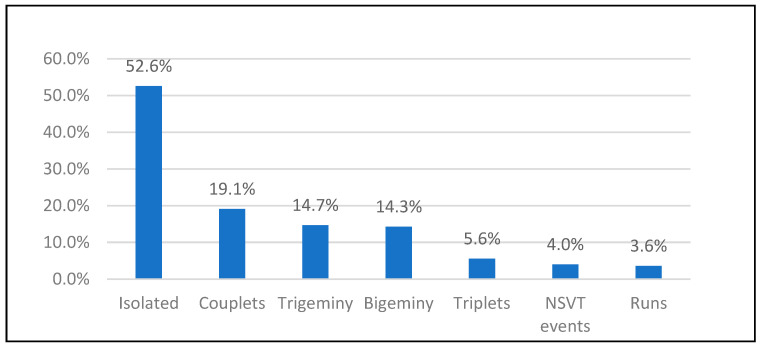
Frequency and pattern distribution of premature ventricular contractions.

PVCs were detected in 134 (53.4%) participants, with median burden 0.0% (IQR 0–0.1%). Most PVCs were isolated (52.6%) or in couplets (19.1%). Higher-grade arrhythmias were uncommon: triplets (5.6%), runs (3.6%), and NSVT (4.0%). Only 4.4% had PVC burden ≥10% (Table 7; Figure 2).

### 3.8. Atrial Ectopy and Supraventricular Arrhythmias

**Figure 3:**
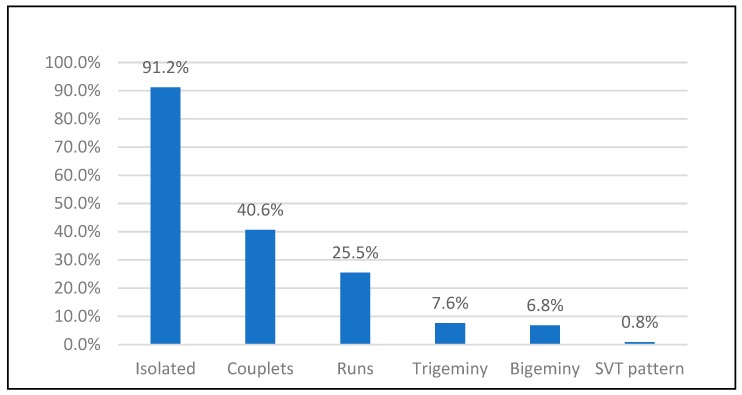
Frequency and pattern distribution of atrial premature contractions and Supraventricular Tachyarrhythmia. SVT, supraventricular contraction.

APCs were highly prevalent (231 participants, 92.0%), with median burden 0.0% (IQR 0–0.1%). Most were isolated (91.2%). APC burden was <5% in 90.8% of participants. SVT and AF each occurred in two patients (0.8%). Sinus bradycardia and tachycardia were frequent (88.8% and 94.8%, respectively) (Table 8 and Table 9; Figure 3).

### 3.9. Symptom Diary Activation

**Figure 4:**
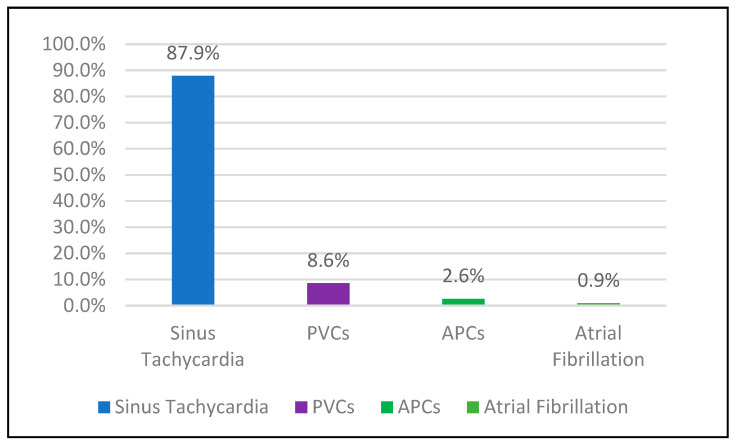
Distribution of cardiac rhythms during symptom diary activation.

Symptom diary was activated by 116 participants (46.2%), recording 996 events. During symptomatic episodes, the rhythm was most commonly sinus tachycardia (87.9%), followed by PVCs (8.6%) and APCs (2.6%). No arrhythmic correlate was found in the remaining 53.8% despite prolonged monitoring (Table 10; Figure 4), indicating that nearly half of patients remain undiagnosed after standard Holter evaluation and may benefit from extended or implantable monitoring strategies.

### 3.10. Correlation Analysis

Correlations between age, mean heart rate, symptom diary entries, and PVC burden were generally weak to modest (Supplementary Figure S3).

### 3.11. Time-Domain Heart Rate Variability

**Figure 5:**
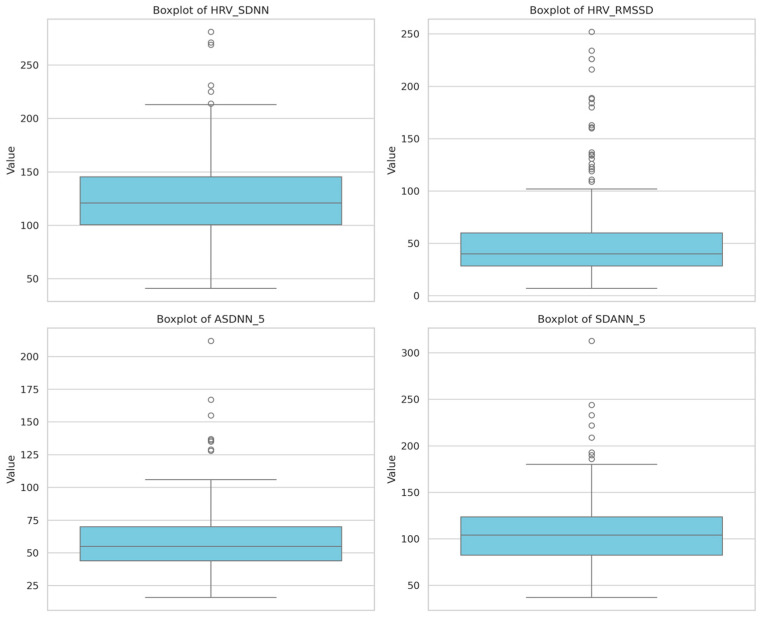
Distribution of time-domain heart rate variability (HRV) parameters derived from 24 to 96 h Holter monitoring, including SDNN, RMSSD, ASDNN, and SDANN.

All time-domain HRV indices exhibited non-normal, right-skewed distributions, indicating marked inter-individual variability in autonomic regulation. SDNN showed the widest dispersion with several high-end outliers. RMSSD values were lower and more tightly clustered, while ASDNN and SDANN demonstrated intermediate dispersion (Figure 5; Supplementary Figures S4 and S5).

These distributional characteristics support the use of non-parametric or distribution-aware analytical approaches for HRV assessment in this population.

### 3.12. Age and Heart Rate Associations with HRV

**Figure 6:**
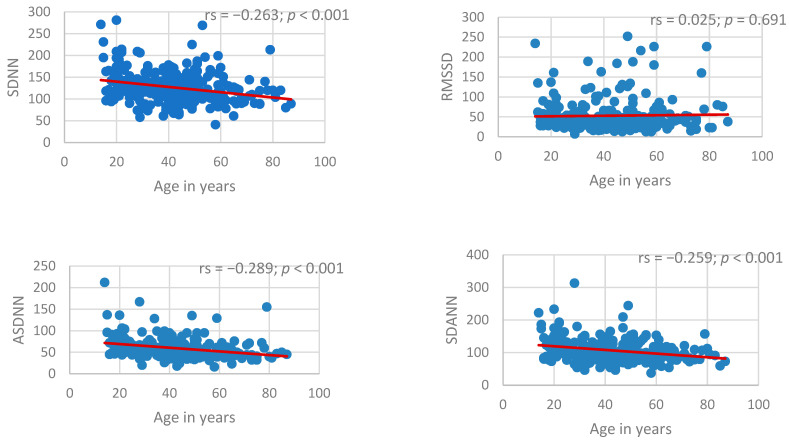
Scatterplots illustrating the relationship between age and time-domain heart rate variability parameters (SDNN, RMSSD, ASDNN, and SDANN).

Age was significantly inversely correlated with SDNN, ASDNN, and SDANN (all *p* < 0.001), indicating an age-related decline in overall and long-term heart rate variability. In contrast, RMSSD showed no significant association with age (*p* = 0.691), suggesting relative preservation of short-term parasympathetic-mediated variability (Figure 6).

**Figure 7:**
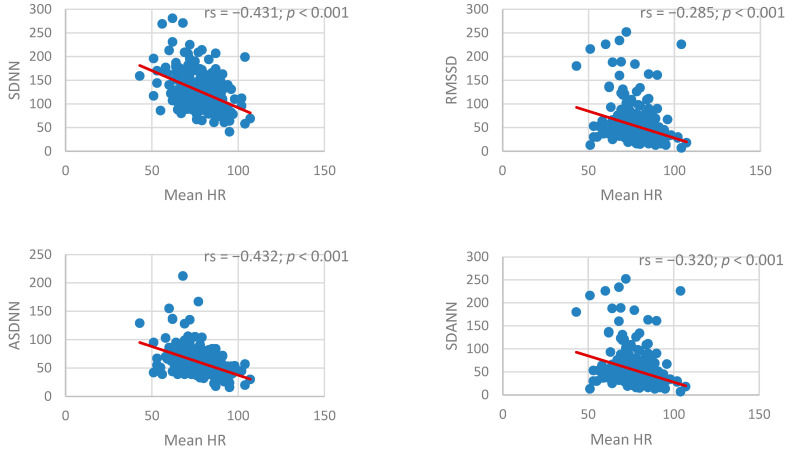
Association between mean heart rate and time-domain heart rate variability indices. HR, heart rate.

Mean heart rate demonstrated significant inverse correlations with all time-domain HRV indices. Higher heart rate was strongly associated with lower SDNN (ρ = −0.431, *p* < 0.001) and ASDNN (ρ = −0.432, *p* < 0.001). Significant negative associations were also observed for RMSSD and SDANN (ρ = −0.285 to −0.320, all *p* < 0.001) (Figure 7).

### 3.13. Psychological Factors and Recording Quality

**Figure 8:**
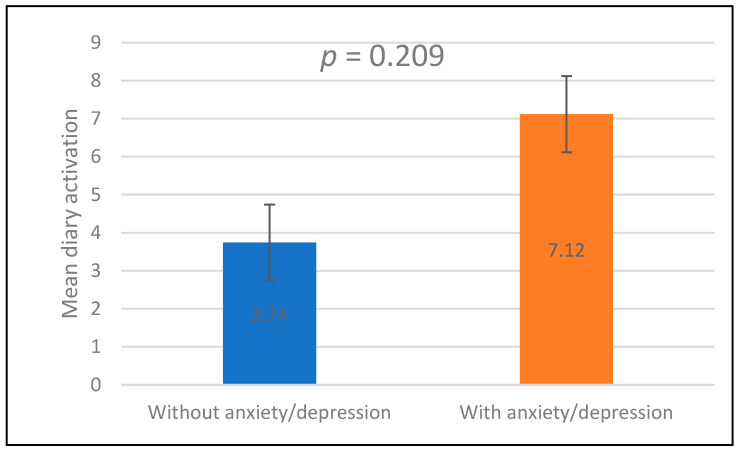
Comparison of symptom diary activation frequency between participants with and without anxiety/depression.

Participants with anxiety/depression had a higher mean number of diary activations compared with those without anxiety/depression (7.12 vs. 3.74). However, this difference was not statistically significant (*p* = 0.209) (Figure 8).

**Figure 9:**
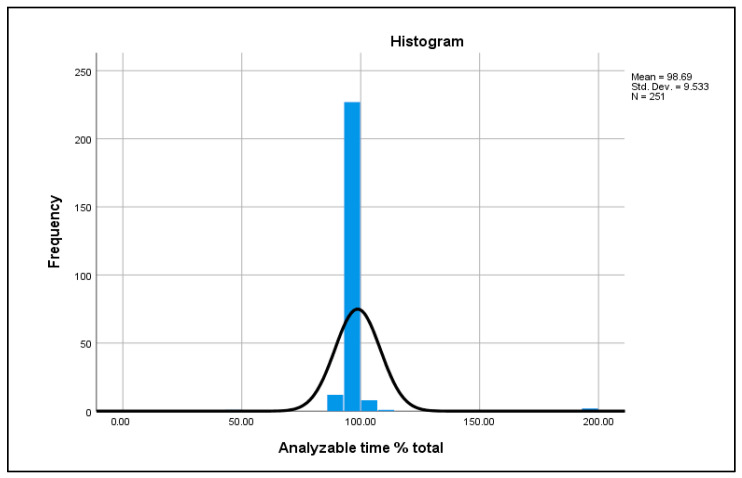
Distribution of analyzable Holter recording time expressed as a percentage of total monitoring duration.

Analyzable recording time was high across the cohort, with a mean of 98.7% (SD ≈ 9.5; n = 251), indicating excellent Holter data quality and minimal signal loss (Figure 9).

### 3.14. Associations of Clinical Characteristics with Ectopy

PVC burden was significantly higher in participants >40 years compared to ≤40 years (1.58 ± 5.08% vs. 0.19 ± 0.64%, *p* = 0.003). No significant associations were found between APC burden and age group, or BMI. PVC burden did not differ significantly by gender, BMI, diabetes, hypertension, or obstructive sleep apnea (Supplementary Tables S2 and S3).

### 3.15. Associations of Beta-Blockers Therapy

Participants on beta-blockers had lower mean heart rate (73.9 ± 10.0 vs. 78.7 ± 10.3 bpm, *p* = 0.007) but significantly higher PVC burden (2.05 ± 5.61% vs. 0.69 ± 3.23%, *p* = 0.031). APC burden was numerically higher but not statistically significant (*p* = 0.157). No significant differences were observed in SVT or bradycardia.

## 4. Discussion

The principal finding of this study is that extended Holter monitoring (24–96 h) provides a diagnostic symptom–rhythm correlation in approximately half (46.2%) of patients presenting with palpitations and structurally normal hearts. This finding reinforces the role of Holter monitoring as an effective first-line screening tool with a diagnostic yield of approximately 50%. Conversely, in the remaining 53.8% of patients, Holter monitoring failed to establish a diagnosis despite extended recording duration, highlighting the need for prolonged monitoring strategies, including external loop recorders or implantable cardiac monitors, to capture infrequent arrhythmias.

From a clinical and health-economic perspective, this stepwise diagnostic approach is particularly relevant. Given that implantable cardiac monitors are associated with substantial costs and resource utilization, the use of extended Holter monitoring as an initial screening tool can effectively identify approximately half of patients without the need for further invasive monitoring. This strategy allows for more targeted use of prolonged monitoring devices in the remaining patients, thereby optimizing diagnostic efficiency while reducing unnecessary healthcare expenditure.

The prevalence of PVCs in our cohort (53.4%) is comparable to previously reported rates in healthy adults undergoing Holter monitoring, where at least one PVC is detected in 69% of individuals [13]. High burdens remain low in our study, consistent with general population data. The use of extended Holter monitoring (24–96 h) likely enhanced detection of intermittent ventricular ectopy [11].

Atrial ectopy was highly prevalent, with APCs detected in over 90% of participants, though burden was generally low. This observation mirrors findings from large cohort studies, demonstrating that atrial premature beats are common in ambulatory monitoring, but only a subset of patients progress to sustained atrial arrhythmias [14]. The low prevalence of AF in our cohort likely reflects the relatively young mean age and preserved atrial size and ventricular function. Nevertheless, APC burden has been associated with future AF and stroke risk in longitudinal studies, underscoring its potential prognostic relevance [8].

Time-domain heart rate variability indices demonstrated marked inter-individual variability and non-normal distributions. Consistent with established autonomic aging patterns, age was inversely correlated with SDNN, ASDNN, and SDANN, reflecting decline in global and long-term autonomic modulation [9]. In contrast, RMSSD was not significantly associated with age, suggesting relative preservation of parasympathetic-mediated short-term variability in this cohort. Similar patterns have been described in population-based HRV studies [15]. Mean heart rate showed robust inverse correlations with all HRV indices, supporting the close interaction between autonomic tone and sinus rate [16].

Despite frequent symptom diary activation, arrhythmia–symptom correlation was limited, with most symptomatic episodes occurring during sinus tachycardia. This finding is consistent with prior reports demonstrating poor concordance between perceived palpitations and documented arrhythmia, particularly in younger and anxious individuals [17]. These observations emphasize the importance of objective rhythm documentation to guide management and avoid unnecessary interventions. Therefore, our findings support a pragmatic diagnostic algorithm in patients with palpitations, where Holter monitoring up to 96 h serves as the initial evaluation step, followed by selective escalation to prolonged external or implantable monitoring in patients without symptom–rhythm correlation.

### Strengths and Limitations

The strengths of this study include its prospective design, high-quality Holter recordings with excellent analyzable time, and comprehensive integration of clinical, echocardiographic, and autonomic data. Limitations include the single-center design and lack of long-term follow-up, precluding assessment of arrhythmia progression or clinical outcomes. Reporting anxiety or depression was identified using documented history in electronic medical records solely, without integrating a validated diagnostic tool. The study was not specifically powered for detailed subgroup analyses; however, the findings remain informative. Larger studies are needed to further validate these findings. Additionally, exclusion of patients with known structural heart disease may limit generalizability to higher-risk populations.

## 5. Conclusions

Extended Holter monitoring (up to 96 h) provides a diagnostic yield in approximately 50% of patients with palpitations and structurally normal hearts, supporting its role as a first-line screening tool. Patients without diagnostic findings may require prolonged monitoring using external or implantable devices.

## Figures and Tables

**Table 1:** Baseline demographic and clinical characteristics (n = 251).

Study Variables	N (%)
Age in years (mean ± SD)	41.9 ± 16.4
≤40 years	120 (47.8%)
>40 years	131 (52.2%)
Male	89 (35.5%)
Female	162 (64.5%)
BMI kg/m^2^ (mean ± SD)	29.5 ± 6.49
BMI ≥ 30 kg/m^2^	113 (45.0%)
Body surface m^2^ (mean ± SD)	1.86 ± 0.24
Systolic blood pressure, mmHg (mean ± SD)	126.4 ± 14.7
Diastolic blood pressure, mmHg (mean ± SD)	76.2 ± 9.76
Currently smoking	12 (4.8%)
Diabetes mellitus	69 (27.5%)
Hypertension	136 (54.2%)
Dyslipidemia	81 (32.3%)
Coronary artery disease	9 (3.6%)
Chronic kidney disease	6 (2.4%)
Obstructive sleep apnea	4 (1.6%)
COPD or asthma	32 (12.7%)
Anemia	35 (13.9%)
Anxiety or depression	17 (6.8%)
Hyperthyroidism	4 (1.6%)
Hypothyroidism	35 (13.9%)

BMI, body mass index; COPD, chronic obstructive pulmonary disease.

**Table 2:** Baseline Laboratory Characteristics.

Variables	Mean ± SD
Hemoglobin, g/dL	13.2 ± 1.78
Thyroid-stimulating hormone (TSH), mIU/L	2.54 ± 3.99
Free thyroxine (FT4), pmol/L	15.6 ± 3.26
Free triiodothyronine (FT3), pmol/L	4.76 ± 1.39
Low-density lipoprotein cholesterol (LDL-C), mmol/L	2.77 ± 0.94
High-density lipoprotein cholesterol (HDL-C), mmol/L	1.39 ± 0.34
Triglycerides, mmol/L	1.19 ± 0.57
Glycated hemoglobin (HbA1c), %	5.83 ± 1.20
Random plasma glucose, mmol/L	6.31 ± 2.48
Fasting plasma glucose, mmol/L	5.89 ± 1.67
Sodium, mmol/L	139.5 ± 2.31
Potassium, mmol/L	4.31 ± 0.37
Calcium, mmol/L	2.34 ± 1.05
Magnesium, mmol/L	0.82 ± 0.09
Creatinine, µmol/L	68.2 ± 21.7

**Table 3:** Baseline transthoracic echocardiographic characteristics.

Parameters	Mean ± SD
Left ventricular ejection fraction (LVEF), %	66.6 ± 5.19
Left ventricular end-diastolic diameter (LVEDD), mm	46.5 ± 4.52
Left atrial anteroposterior diameter, mm	30.7 ± 4.88
Interventricular septal thickness, mm	8.09 ± 1.31
Left ventricular posterior wall thickness, mm	7.63 ± 1.49
Tricuspid annular plane systolic excursion (TAPSE), mm	21.6 ± 2.37

**Table 4:** Baseline Medical Therapy.

Medication	N (%)
Beta-blockers	42 (16.7%)
Statins	55 (21.9%)
ACE inhibitors or ARBs	24 (9.6%)
Diuretics	9 (3.6%)
Oral anticoagulants	6 (2.4%)

ACE, angiotensin-converting enzyme inhibitors; ARBs, angiotensin receptor blockers.

**Table 5:** Holter monitoring quality and heart rate characteristics.

Parameter	Mean ± SD
Analyzable recording time, h	43.5 ± 15.2
Analyzable time, % of total	98.7 ± 9.53
Total QRS complexes	194,525 ± 71,330
Maximum heart rate, bpm	135.2 ± 21.4
Minimum heart rate, bpm	51.8 ± 8.56
Mean heart rate, bpm	77.9 ± 10.4
Monitoring duration	N (%)
24 h	69 (27.5%)
48 h	157 (62.5%)
72 h	21 (8.4%)
96 h	4 (1.6%)

bpm, beats per minute.

**Table 6:** Time-domain heart rate variability parameters.

Heart Rate Variability Parameter	Mean ± SD
SDNN, ms	126.6 ± 37.8
RMSSD, ms	53.0 ± 41.7
ASDNN (5 min), ms	59.9 ± 24.3
SDANN (5 min), ms	107.0 ± 35.7

ASDNN, average of the standard deviations of all normal-to-normal intervals; SDANN, standard deviation of the average normal-to-normal intervals; SDNN, standard deviation of normal-to-normal intervals; RMSSD, root mean square of successive differences.

**Table 7:** Characteristics of premature ventricular contractions (n = 251).

PVC Presence and Burden	N (%)
PVC present	134 (53.4%)
PVC count, median (range)	1 (0–64,826)
PVC burden, %, median (IQR)	0.0 (0–0.1)
PVC morphology (n = 134)	
One morphology	119 (88.8%)
Two morphologies	15 (11.2%)
PVC types (not mutually exclusive; % of total cohort)	
Isolated PVCs	132 (52.6%)
PVC Bigeminy	36 (14.3%)
PVC Trigeminy	37 (14.7%)
PVC Couplets	48 (19.1%)
PVC Triplets	14 (5.6%)
PVC Runs	9 (3.6%)
NSVT events	10 (4.0%)
NSVT duration (s), median (range)	2.5 (2.0–5.0)
PVC burden categories	
None	117 (46.6%)
<1–5%	123 (49.0%)
6–10%	2 (0.80%)
11–15%	4 (1.6%)
16–20%	3 (1.2%)
>20%	2 (0.80%)

IQR, interquartile range; PVC, premature ventricular contraction; NSVT, nonsustained ventricular tachycardia.

**Table 8:** Characteristics of atrial premature contractions (n = 251).

APC Presence and Burden	N (%)
APCs present	231 (92.0%)
APC Count, median (range)	40 (1–27,870)
APC Burden %, median (IQR)	0.0 (0–0.1)
APC patterns (not mutually exclusive; % of total cohort)	
APC Pattern Isolated	229 (91.2%)
APC Pattern Bigeminy	17 (6.8%)
APC Pattern Trigeminy	19 (7.6%)
APC Pattern Couplets	102 (40.6%)
APC Pattern Runs	64 (25.5%)
Runs Total Beats, median (range)	0.0 (0–169)
APC burden categories	
None	20 (8.0%)
<1–5%	228 (90.8%)
6–10%	1 (0.40%)
11–15%	1 (0.40%)
>15%	1 (0.40%)

APC, atrial premature contractions.

**Table 9:** Supraventricular arrhythmias and Sinus Rhythm Abnormalities (n = 251).

Parameter	N (%)
Supraventricular tachycardia (SVT)	2 (0.80%)
Atrial fibrillation (AF)	2 (0.80%)
AF burden (%), median (min–max)	15.5 (2.2–28.7)
Sinus bradycardia	223 (88.8%)
Sinus tachycardia	238 (94.8%)
Symptom diary activation	116 (46.2%)

**Table 10:** Patient-reported symptoms and diary activation (n = 251).

Parameter	N (%)
Patients activating symptom diary	116 (46.2%)
Diary events recorded	996
Tachycardia Type during Diary Events (n = 116)	
Sinus tachycardia	102 (87.9%)
PVCs	10 (8.6%)
APCs	3 (2.6%)
Atrial fibrillation	1 (0.90%)

## Data Availability

The datasets used and/or analyzed during the current study are available from the corresponding author upon reasonable request.

## Supplementary Materials

The following supporting information can be downloaded at: https://www.mdpi.com/article/10.3390/jcm15093285/s1, Figure S1: Distribution of premature ventricular contraction (PVC) burden across the study cohort; Figure S2: Distribution of atrial premature contraction (APC) burden across the study cohort; Figure S3: Correlation heatmap of age, mean heart rate, symptom diary entries, and PVC burden; Figure S4: Violin plots illustrating the distribution and variability of time-domain heart rate variability indices; Figure S5: Histograms demonstrating right-skewed distributions of time-domain HRV measures; Table S1: Definitions of Holter Monitoring Parameters Used in the Study; Table S2: Association between APC burden, age, hypertension, and BMI level (n = 251); Table S3: Association between PVC burden and the demographic and clinical characteristics (n = 251); Table S4: Association between Beta-blockers therapy and ambulatory ECG findings; Table S5: Association between the most comorbidities and mean heart rate (n = 251); Table S6: Association between APC burden, Heart rhythm, and comorbidities (n = 251); Table S7: Association between PVC burden, Heart rhythm, and comorbidities (n = 251). References [18,19,20,21,22,23,24,25,26,27] are cited in the Supplementary Materials

## Abbreviations

The following abbreviations are used in this manuscript:

ACE	Angiotensin-converting enzyme
AF	Atrial fibrillation
APCs	Atrial premature contractions
ARB	Angiotensin receptor blocker
ASDNN	Average of the standard deviations of all normal-to-normal intervals
AV	Atrioventricular block
AVNRT	Atrioventricular nodal reentrant tachycardia
AVRT	Atrioventricular reentrant tachycardia
BMI	Body mass index
BPM	Beats per minute
ECG	Electrocardiogram
FT3	Free triiodothyronine
FT4	Free thyroxine
HbA1c	Glycated hemoglobin
HDL-C	High-density lipoprotein cholesterol
HRV	Heart rate variability
IQR	Interquartile range
LBBB	Left bundle branch block
LDL-C	Low-density lipoprotein cholesterol
LVEDD	Left ventricular end-diastolic diameter
LVEF	Left ventricular ejection fraction
NSVT	Nonsustained ventricular tachycardia
PACs	Premature atrial contractions
PVCs	Premature ventricular contractions
RMSSD	Root mean square of successive differences
SDANN	Standard deviation of the average normal-to-normal intervals
SDNN	Standard deviation of normal-to-normal intervals
STROBE	Strengthening the Reporting of Observational Studies in Epidemiology
SVT	Supraventricular tachycardia
TAPSE	Tricuspid annular plane systolic excursion
TSH	Thyroid-stimulating hormone
VF	Ventricular fibrillation
VT	Ventricular tachycardia
