# Supplementary material for: Ambulatory Holter Findings in Patients with Palpitations and Structurally Normal Heart: A Prospective Study of the Prevalence and Patterns of Ventricular and Supraventricular Arrhythmias
Source: J Clin Med. 2026 Apr 25;15(9):3285. doi: 10.3390/jcm15093285 (PMC13163502; doi:10.3390/jcm15093285)
Supplement: Supplementary file 1 [file jcm-15-03285-s001.zip › jcm-4231506-supplementary.pdf]

Table S1. Definitions of Holter Monitoring Parameters Used in the Study.

Parameter Category	Parameter	Definition
**Heart Rate Variability (HRV)**	SDNN	Standard deviation of all NN intervals over 24 hours; reflects overall autonomic modulation.[9]
	RMSSD	Square root of the mean squared differences between adjacent NN intervals; reflects parasympathetic tone.[9]
	ASDNN	ASDNN5 stands for the average of the standard deviations of the N-N (normal-to-normal) intervals calculated over consecutive 5-minute segments of a long Holter electrocardiogram recording.[18,19]
	SDANN	SD of the average NN intervals for all 5-minute segments reflects long-term HRV components.[18,19]
**Premature Ventricular Complexes (PVCs)**	PVC Count	Total number of PVCs recorded during a specified electrocardiographic monitoring period.[9,20]
	PVC Burden (%)	PVCs as % of all beats; frequent ≥5%, high ≥10%.[9,20,21]
	PVC Morphology	Classification by QRS shape and pattern (unifocal, multifocal, LBBB-type, RBBB-type).[9,20,21]
	Isolated PVC	Single PVC separated from others and independent.[9]
	Bigeminy	Every normal sinus beat is followed by a premature ventricular contraction (PVC).[20,22]
	Trigeminy	Every two normal sinus beat is followed by a premature ventricular contraction (PVC).[20,22]
	Couplets	Two consecutive PVCs with no normal beats in between.[20,22]
	Triplets	Three consecutive PVCs with no normal beats in between. [20,22]
	PVC Runs	≥4 consecutive PVCs (ventricular tachyarrhythmia).[20,22]
**Ventricular Tachyarrhythmias**	NSVT	≥3 consecutive PVCs at >100 bpm lasting <30 s (spontaneously terminating).[20,23,24]
	Sustained VT	VT lasting ≥30 s or requiring termination for instability.[20,23,24]
**Premature Atrial Contractions (PAC/APC)**	APC Count	Total number of PACs recorded during Holter monitoring study.[20,22]
	APC Burden (%)	PACs as % of all recorded beats.[20,22,25]
	Isolated APC	Single premature atrial beat separated and independent of others.[20,22,25]

	APC Bigeminy/Trigeminy	Repetitive PACs every 2nd or 3rd beat.[20,22]
	APC Couplets	Two consecutive PACs and normal rhythm afterwards.[20,22]
	APC Runs / Atrial Runs	≥3 consecutive PACs (short atrial tachycardia).[20,22]
	Runs — Total Beats	Total atrial ectopic beats within all runs.[20,22]
**Supraventricular Tachyarrhythmias**	SVT Count	Number of SVT episodes.[20,22,26]
	SVT Type	Atrial tachycardia, AVNRT, AVRT, or unclassified.[20,26]
	SVT Duration	Duration of each SVT episode (s).[20,26]
**Atrial Fibrillation / Flutter**	AF Presence	Any AF detected during monitoring.[20,27]
	AF Burden (%)	% of total monitoring time spent in AF.[20,27]
	Atrial Flutter Presence	Typical or atypical flutter documented.[20,27]
	AT Count	Number of atrial tachycardia episodes independent of AF/flutter.[20,27]
**Other Arrhythmias**	Junctional Ectopy	Premature QRS complexes originating from atrioventricular junction & absent p wave.[20,22,23]
	Sinus Bradycardia	Sinus rate <60 bpm (clinically relevant <50 bpm).[20,22,23]
**Pauses / Conduction Disorders**	Pause Count	Number of sinus pauses of atrial depolarization, resulting in a pause in ventricular activity (missing QRS complex).[20,22,23]
	Longest Pause	Longest pause (seconds).[20,22,23]
	Symptomatic Pause	Pause temporally linked to symptoms.[20,22,23]
	1st-degree AV Block	PR interval >200 ms.[20,23]
	2nd-degree AV Block	Mobitz I or Mobitz II patterns.[20,23]
	3rd-degree AV Block	Complete AV dissociation.[20,23]
**Sinus Tachycardia**	Sinus Tachycardia	Sinus rhythm >100 bpm.[20,22,23]
**Malignant Ventricular Rhythms**	Ventricular Fibrillation	VF episodes detected during Holter.[20,22,23]
	Torsades de Pointes	Polymorphic VT with QT prolongation and axis twisting.[20,22,23]
**Symptom Correlation**	Diary Symptoms	Patient-reported symptoms during Holter period.[20,22]
	Diary Tachycardia	Symptoms correlating with documented tachyarrhythmia. [20,22]

AV, atrioventricular block; AVNRT, atrioventricular nodal reentrant tachycardia; AVRT, atrioventricular reentrant tachycardia; LBBB, left bundle branch block; PAC, premature atrial complex; RBBB, right bundle branch block; VF, ventricular fibrillation; VT, ventricular tachycardia. Other abbreviations as defined in previous tables and figures.

Table S2. Association between APC burden, age, hypertension, and BMI level (n=251).

Variable	APC burden		P-value §
	No<br>N (%)<br>(n=20)	Yes<br>N (%)<br>(n=231)	
Age group			
• ≤40 years	08 (40.0%)	112 (48.5%)	0.466
• >40 years	12 (60.0%)	119 (51.5%)	
Hypertension			
• No	10 (50.0%)	128 (55.4%)	0.641
• Yes	10 (50.0%)	103 (44.6%)	
BMI level			
• <30 kg/m2	15 (75.0%)	157 (68.0%)	0.516
• ≥30 kg/m2	05 (25.0%)	74 (32.0%)	

§ P-value has been calculated using Chi-square test.

Table S3. Association between PVC burden and the demographic and clinical characteristics (n=251).

Study variables	PVC Burden (%)	t-test	P-value §
Age group			
• ≤40 years	0.19 ± 0.64	-2.986	**0.003** **
• >40 years	1.58 ± 5.08		
Gender			
• Male	1.46 ± 4.60	1.699	0.090
• Female	0.62 ± 3.17		
BMI level			
• BMI <30 kg/m2	0.73 ± 3.39	-0.853	0.394
• BMI ≥30 kg/m^2	1.14 ± 4.15		
Diabetes mellitus			
• No	0.66 ± 3.13	-1.794	0.074
• Yes	1.60 ± 5.01		
HbA1c level			
• ≤7.5%	1.11 ± 4.34	-0.294	0.769
• >7.5%	1.44 ± 3.33		
Obstructive sleep apnea			
• No	0.93 ± 3.78	0.345	0.731
• Yes	0.27 ± 0.50		

§ P-value has been calculated using independent sample t-test.

** Significant at p<0.05 level.

Table S4. Association between Beta-blockers therapy and ambulatory ECG findings.

Variable	Beta-blockers therapy No Mean ± SD	Beta-blockers therapy Yes Mean ± SD	P-value §
Mean heart rate	78.7 ± 10.3	73.9 ± 10.0	**0.007** **
PVC burden (%)	0.69 ± 3.23	2.05 ± 5.61	**0.031** **
APC burden (%)	0.18 ± 0.87	0.49 ± 2.56	0.157
Presence of SVT, N (%)			
• No	208 (99.5%)	41 (97.6%)	0.307
• Yes	1 (0.5%)	1 (2.4%)	
Presence of bradycardia			
• No	23 (11.0%)	5 (11.9%)	0.793
• Yes	186 (89.0%)	37 (88.1%)	

§ P-value has been calculated using independent sample t-test.

** Significant at p<0.05 level.

Table S5. Association between the most comorbidities and mean heart rate (n=251).

Study variables	Mean HR	t-test	P-value §
Diabetes mellitus			
• No	78.1 ± 10.6	0.445	0.657
• Yes	77.4 ± 9.94		
Hypertension			
• No	78.8 ± 10.1	1.991	**0.048** **
• Yes	75.9 ± 10.9		
Dyslipidemia			
• No	78.7 ± 10.5	1.843	0.067
• Yes	76.1 ± 10.0		
COPD asthma			
• No	77.7 ± 10.5	0.877	0.382
• Yes	79.4 ± 9.75		
Anxiety and depression			
• No	77.9 ± 10.4	0.439	0.661
• Yes	76.8 ± 11.5		
Hypothyroidism			
• No	78.1 ± 10.1	0.757	0.450
• Yes	76.7 ± 12.3		

§ P-value has been calculated using independent sample t-test.

** Significant at p<0.05 level.

Table S6. Association between APC burden, Heart rhythm, and comorbidities (n=251).

Variable	APC burden No N (%) (n=20)	APC burden Yes N (%) (n=231)	P-value §
Diabetes			
• No	14 (70.0%)	168 (72.7%)	0.793
• Yes	6 (30.0%)	63 (27.3%)	
Hypertension			

• No	15 (75.0%)	157 (68.0%)	0.516
• Yes	5 (25.0%)	74 (32.0%)	
Dyslipidemia			
• No	12 (60.0%)	158 (68.4%)	0.441
• Yes	8 (40.0%)	73 (31.6%)	
COPD asthma			
• No	15 (75.0%)	204 (88.3%)	0.087
• Yes	5 (25.0%)	27 (11.7%)	
Anxiety and depression			
• No	18 (90.0%)	216 (93.5%)	0.633
• Yes	2 (10.0%)	15 (6.5%)	
Hypothyroidism			
• No	19 (95.0%)	197 (85.3%)	0.325
• Yes	1 (5.0%)	34 (14.7%)	
SDNN (mean ± SD) ‡	137.8 ± 33.5	125.6 ± 38.1	0.170
RMSSD (mean ± SD) ‡	89.3 ± 65.8	49.9 ± 37.5	**<0.001** **
ASDNN (mean ± SD) ‡	68.2 ± 33.5	59.1 ± 23.3	0.112
SADNN (mean ± SD) ‡	116.2 ± 54.8	106.2 ± 33.6	0.232

§ P-value has been calculated using Chi-square test.

‡ P-value has been calculated using independent sample t-test.

** Significant at p<0.05 level.

Table S7. Association between PVC burden, Heart rhythm, and comorbidities (n=251).

**Variable**	**PVC burden**		**P-value** §
	**No** **N (%)** (n=117)	**Yes** **N (%)** (n=134)	
Diabetes			
• No	98 (83.8%)	84 (62.7%)	**<0.001** **
• Yes	19 (16.2%)	50 (37.3%)	
Hypertension			
• No	94 (80.3%)	78 (58.2%)	**<0.001** **
• Yes	23 (19.7%)	56 (41.8%)	
Dyslipidemia			
• No	97 (82.9%)	73 (54.5%)	**<0.001** **
• Yes	20 (17.1%)	61 (45.5%)	
COPD asthma			
• No	100 (85.5%)	119 (88.8%)	0.429
• Yes	17 (14.5%)	15 (11.2%)	
Anxiety and depression			
• No	111 (94.9%)	123 (91.8%)	0.333
• Yes	6 (5.1%)	11 (8.2%)	
Hypothyroidism			
• No	105 (89.7%)	111 (82.8%)	0.115
• Yes	12 (10.3%)	23 (17.2%)	
SDNN (mean ± SD) ‡	131.3 ± 39.3	122.5 ± 36.2	0.067
RMSSD (mean ± SD) ‡	47.9 ± 32.9	57.5 ± 47.6	0.068
ASDNN (mean ± SD) ‡	61.5 ± 27.2	58.4 ± 21.4	0.306
SADNN (mean ± SD) ‡	114.7 ± 39.9	100.4 ± 30.0	**0.001** **

§ P-value has been calculated using Chi-square test.

‡ P-value has been calculated using independent sample t-test.

** Significant at p<0.05 level.

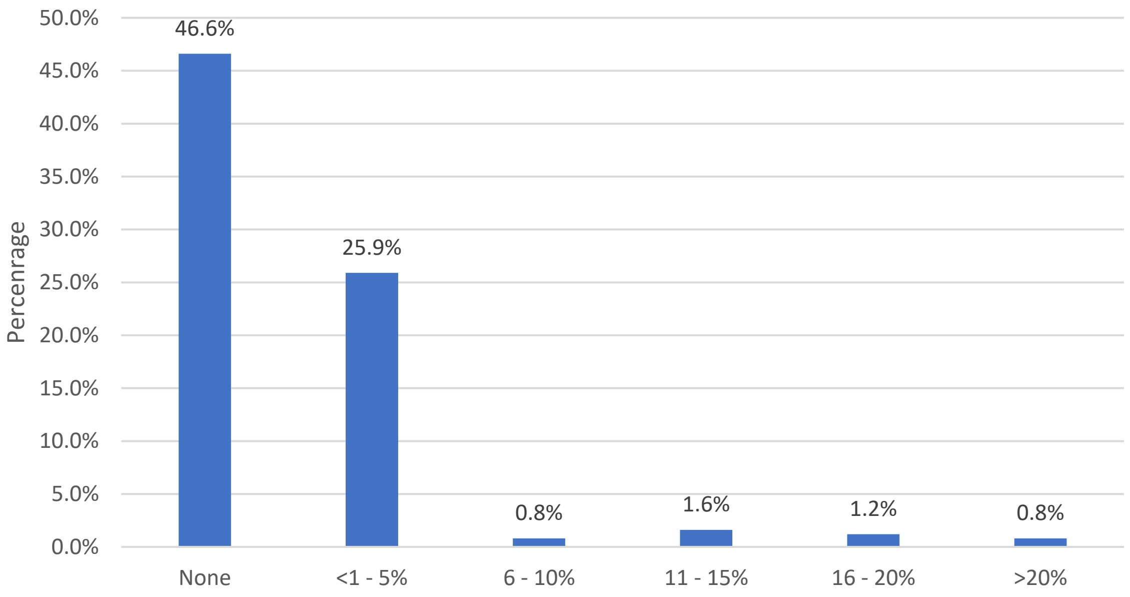

Figure S1. Distribution of premature ventricular contraction (PVC) burden across the study cohort.

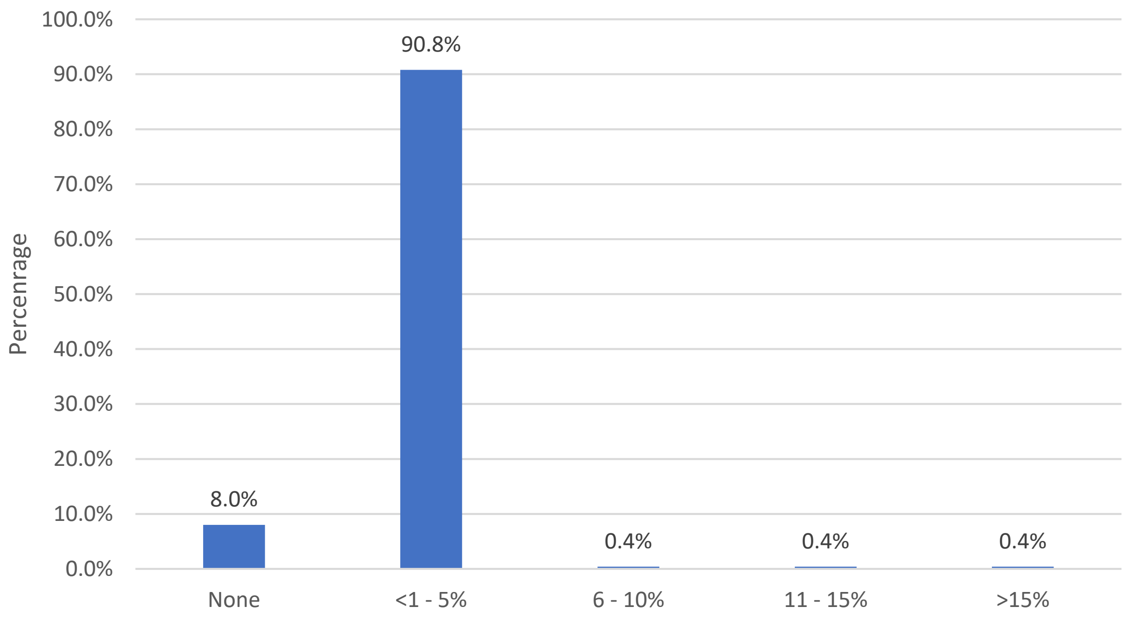

Figure S2. Distribution of atrial premature contraction (APC) burden across the study cohort.

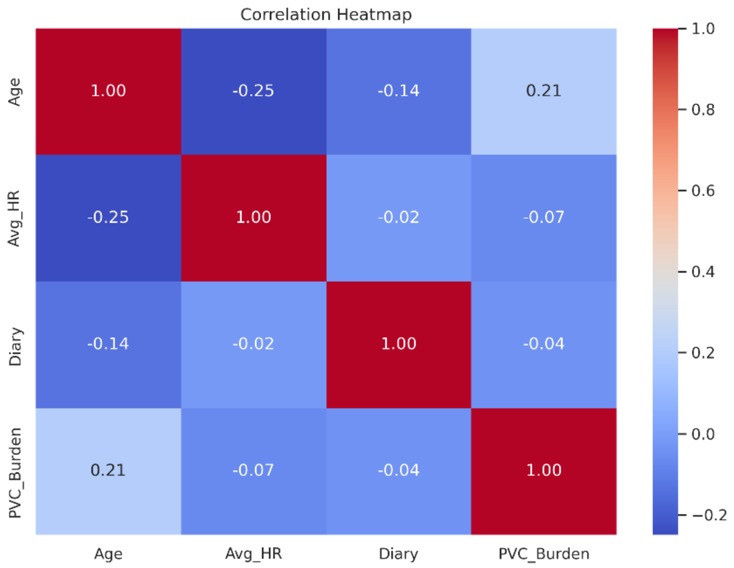

Figure S3. Correlation heatmap of age, mean heart rate, symptom diary entries, and PVC burden.

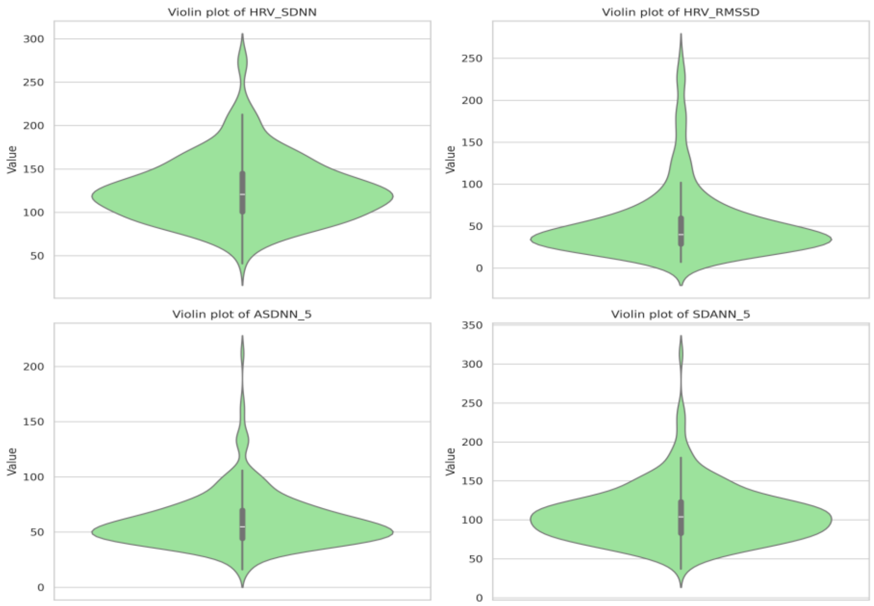

Figure S4. Violin plots illustrating the distribution and variability of time-domain heart rate variability indices.

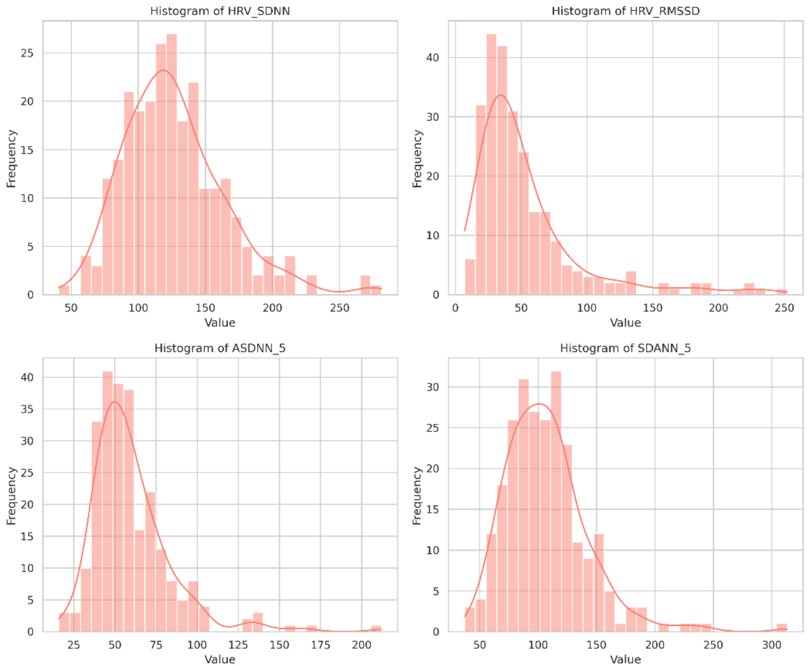

Figure S5. Histograms demonstrating right-skewed distributions of time-domain HRV measures.

References

1. Govender, I.; Nashed, K.K.; Rangiah, S.; Okeke, S.; Maphasha, O.M. Palpitations: Evaluation and management by primary care practitioners. *S. Afr. Fam. Pract.* **2022**, *64*, e1–e8. https://doi.org/10.4102/safp.v64i1.5449.
2. Crawford, M.H.; Bernstein, S.J.; Deedwania, P.C.; DiMarco, J.P.; Ferrick, K.J.; Garson, A., Jr.; Green, L.A.; Greene, H.L.; Silka, M.J.; Stone, P.H.; et al. ACC/AHA Guidelines for Ambulatory Electrocardiography: A report of the American College of Cardiology/American Heart Association Task Force on Practice Guidelines (Committee to Revise the Guidelines for Ambulatory Electrocardiography). Developed in collaboration with the North American Society for Pacing and Electrophysiology. *J. Am. Coll. Cardiol.* **1999**, *34*, 912–948. https://doi.org/10.1016/s0735-1097(99)00354-x.
3. Priori, S.G.; Blomström-Lundqvist, C.; Mazzanti, A.; Blom, N.; Borggrefe, M.; Camm, J.; Elliott, P.M.; Fitzsimons, D.; Hatala, R.; Hindricks, G.; et al. 2015 ESC Guidelines for the management of patients with ventricular arrhythmias and the prevention of sudden cardiac death: The Task Force for the Management of Patients with Ventricular Arrhythmias and the Prevention of Sudden Cardiac Death of the European Society of Cardiology (ESC). Endorsed by: Association for European Paediatric and Congenital Cardiology (AEPC). *Eur. Heart J.* **2015**, *36*, 2793–2867. https://doi.org/10.1093/eurheartj/ehv316.
4. Tsiachris, D.; Botis, M.; Doundoulakis, I.; Bartsioka, L.I.; Tsioufis, P.; Kordalis, A.; Antoniou, C.-K.; Tsioufis, K.; Gatzoulis, K.A. Electrocardiographic Characteristics, Identification, and Management of Frequent Premature Ventricular Contractions. *Diagnostics* **2023**, *13*, 3094. https://doi.org/10.3390/diagnostics13193094.
5. Ahn, M.S. Current Concepts of Premature Ventricular Contractions. *J. Lifestyle Med.* **2013**, *3*, 26–33.
6. Marcus, G.M. Evaluation and Management of Premature Ventricular Complexes. *Circulation* **2020**, *141*, 1404–1418. https://doi.org/10.1161/CIRCULATIONAHA.119.042434.
7. Conen, D.; Adam, M.; Roche, F.; Barthelemy, J.-C.; Dietrich, D.F.; Imboden, M.; Künzli, N.; von Eckardstein, A.; Regenass, S.; Hornemann, T.; et al. Premature atrial contractions in the general population: Frequency and risk factors. *Circulation* **2012**, *126*, 2302–2308. https://doi.org/10.1161/CIRCULATIONAHA.112.112300.
8. Larsen, B.S.; Kumarathurai, P.; Falkenberg, J.; Nielsen, O.W.; Sajadieh, A. Excessive Atrial Ectopy and Short Atrial Runs Increase the Risk of Stroke Beyond Incident Atrial Fibrillation. *J. Am. Coll. Cardiol.* **2015**, *66*, 232–241. https://doi.org/10.1016/j.jacc.2015.05.018.
9. Task Force of the European Society of Cardiology and the North American Society of Pacing and Electrophysiology. Heart rate variability: Standards of measurement, physiological interpretation and clinical use. *Circulation* **1996**, *93*, 1043–1065.

10. Chua, S.-K.; Chen, L.-C.; Lien, L.-M.; Lo, H.-M.; Liao, Z.-Y.; Chao, S.-P.; Chuang, C.-Y.; Chiu, C.-Z. Comparison of Arrhythmia Detection by 24-Hour Holter and 14-Day Continuous Electrocardiography Patch Monitoring. *Acta Cardiol. Sin.* **2020**, *36*, 251–259. https://doi.org/10.6515/ACS.202005_36(3).20190903A.
11. Zimetbaum, P.; Goldman, A. Ambulatory arrhythmia monitoring: Choosing the right device. *Circulation* **2010**, *122*, 1629–1636. https://doi.org/10.1161/CIRCULATIONAHA.109.925610.
12. von Elm, E.; Altman, D.G.; Egger, M.; Pocock, S.J.; Gotzsche, P.C.; Vandenbroucke, J.P. Strengthening the Reporting of Observational Studies in Epidemiology (STROBE) statement: Guidelines for reporting observational studies. *BMJ* **2007**, *335*, 806–808. https://doi.org/10.1136/bmj.39335.541782.AD.
13. von Rotz, M.; Aeschbacher, S.; Bossard, M.; Schoen, T.; Blum, S.; Schneider, S.; Estis, J.; Todd, J.; Risch, M.; Risch, L.; et al. Risk factors for premature ventricular contractions in young and healthy adults. *Heart* **2017**, *103*, 702–707. https://doi.org/10.1136/heartjnl-2016-309632.
14. Guichard, J.B.; Guasch, E.; Roche, F.; Da Costa, A.; Mont, L. Premature atrial contractions: A predictor of atrial fibrillation and a relevant marker of atrial cardiomyopathy. *Front. Physiol*. **2022**, *13*, 971691. https://doi.org/10.3389/fphys.2022.971691.
15. Reardon, M.; Malik, M. Changes in heart rate variability with age. *Pacing Clin. Electrophysiol.* **1996**, *19*, 1863–1866. https://doi.org/10.1111/j.1540-8159.1996.tb03241.x.
16. Kazmi, S.Z.; Zhang, H.; Aziz, W.; Monfredi, O.; Abbas, S.A.; Shah, S.A.; Kazmi, S.S.H.; Butt, W.H. Inverse Correlation between Heart Rate Variability and Heart Rate Demonstrated by Linear and Nonlinear Analysis. *PLoS ONE* **2016**, *11*, e0157557. https://doi.org/10.1371/journal.pone.0157557.
17. Sulfi, S.; Balami, D.; Sekhri, N.; Suliman, A.; Kapur, A.; Archbold, R.A.; Ranjadayalan, K.; Timmis, A.D. Limited clinical utility of Holter monitoring in patients with palpitations or altered consciousness: Analysis of 8973 recordings in 7394 patients. *Ann. Noninvasive Electrocardiol.* **2008**, *13*, 39–43. https://doi.org/10.1111/j.1542-474X.2007.00199.x.
18. Kamalesh, M.; Burger, A.J.; Kumar, S.; Nesto, R. Reproducibility of time and frequency domain analysis of heart rate variability in patients with chronic stable angina. *Pacing Clin. Electrophysiol*. **1995**, *18*, 1991–1994. https://doi.org/10.1111/j.1540-8159.1995.tb03858.x.
19. Zandstra, T.; Kiès, P.; Maan, A.; Man, S.-C.; Bootsma, M.; Vliegen, H.; Egorova, A.; Mertens, B.; Holman, E.; Schalij, M.; et al. Association between reduced heart rate variability components and supraventricular tachyarrhythmias in patients with a systemic right ventricle. *Auton. Neurosci.* **2020**, *227*, 102696. https://doi.org/10.1016/j.autneu.2020.102696.
20. Zipes, D.P.; Jalife, J.; Stevenson, W.G. *Cardiac Electrophysiology: From Cell to Bedside*, 7th ed.; Elsevier: Amsterdam, The Netherlands, 2018.
21. Baman, T.S.; Lange, D.C.; Ilg, K.J.; Gupta, S.K.; Liu, T.-Y.; Alguire, C.; Armstrong, W.; Good, E.; Chugh, A.; Jongnarangsin, K.; et al. Relationship between burden of premature ventricular complexes and left ventricular function. *Heart Rhythm* **2010**, *7*, 865–869. https://doi.org/10.1016/j.hrthm.2010.03.036.
22. Goldberger, A.L.; Goldberger, Z.D.; Shvilkin, A. *Goldberger's Clinical Electrocardiography: A Simplified Approach*, 9th ed.; Elsevier: Amsterdam, The Netherlands, 2018.
23. Al-Khatib, S.M.; Stevenson, W.G.; Ackerman, M.J.; Bryant, W.J.; Callans, D.J.; Curtis, A.B.; Deal, B.J.; Dickfeld, T.; Field, M.E.; Fonarow, G.C.; et al. 2017 AHA/ACC/HRS guideline for management of patients with ventricular arrhythmias and the prevention of sudden cardiac death. *Circulation* **2018**, *138, e272-e391.* https://doi.org/10.1161/cir.0000000000000549.
24. Zeppenfeld, K.; Tfelt-Hansen, J.; de Riva, M.; Winkel, B.G.; Behr, E.R.; A Blom, N.; Charron, P.; Corrado, D.; Dagres, N.; de Chillou, C.; et al. 2022 ESC Guidelines for the management of patients with ventricular arrhythmias and the prevention of sudden cardiac death. *Eur. Heart J*. **2022**, *43*, 3997–4126. https://doi.org/10.1093/eurheartj/ehac262.
25. Binici, Z.; Intzilakis, T.; Nielsen, O.W.; Køber, L.; Sajadieh, A. Excessive supraventricular ectopic activity and increased risk of atrial fibrillation and stroke. *Circulation* **2010**, *121*, 1904–1911. https://doi.org/10.1161/CIRCULATIONAHA.109.874982.
26. Brugada, J.; Katritsis, D.G.; Arbelo, E.; Arribas, F.; Bax, J.J.; Blomström-Lundqvist, C.; Calkins, H.; Corrado, D.; Deftereos, S.G.; Diller, G.-P.; et al. 2019 ESC Guidelines for the management of patients with supraventricular tachycardiaThe Task Force for the management of patients with supraventricular tachycardia of the European Society of Cardiology (ESC). *Eur. Heart J*. **2020**, *41*, 655–720. https://doi.org/10.1093/eurheartj/ehz467.
27. Wann, L.S.; Curtis, A.B.; January, C.T.; Ellenbogen, K.A.; Lowe, J.E.; Estes, I.N.M.; Page, R.L.; Ezekowitz, M.D.; Slotwiner, D.J.; Jackman, W.M.; et al. 2011 ACCF/AHA/HRS focused update on the management of patients with atrial fibrillation (updating the 2006 guideline). *Circulation* **2011**, *123*, 104–123. https://doi.org/10.1161/cir.0b013e3181fa3cf4.
